# Rapamycin Improves Adipose-Derived Mesenchymal Stem Cells (ADMSCs) Renoprotective Effect against Cisplatin-Induced Acute Nephrotoxicity in Rats by Inhibiting the mTOR/AKT Signaling Pathway

**DOI:** 10.3390/biomedicines10061295

**Published:** 2022-05-31

**Authors:** Amira Awadalla, Abdelaziz M. Hussein, Yousra M. El-Far, Fardous F. El-Senduny, Nashwa Barakat, Eman T. Hamam, Hanaa M. Abdeen, Mohamed El-Sherbiny, Mohamed S. Serria, Amira A. Sarhan, Asmaa M. Sena, Ahmed A. Shokeir

**Affiliations:** 1 Center of Excellence for Genome and Cancer Research, Urology and Nephrology Center, Mansoura University, Mansoura 35516, Egypt; a.lahlouba@hotmail.com (A.A.); nashwab2006@yahoo.com (N.B.); et.hamam@hotmail.com (E.T.H.); amiralsarhan@gmail.com (A.A.S.); asmaamahmoud2022@gmail.com (A.M.S.); ahmed.shokeir@hotmail.com (A.A.S.); 2 Medical Physiology Department, Faculty of Medicine, Mansoura University, Mansoura 35516, Egypt; 3 Department of Biochemistry, Faculty of Pharmacy, Mansoura University, Mansoura 35516, Egypt; yousraelfar@yahoo.com; 4 Biochemistry Division, Chemistry Department, Faculty of Sciences, Mansoura University, Mansoura 35516, Egypt; fkaneer@mans.edu.eg; 5 Medical Biochemistry and Molecular Biology Department, Faculty of Medicine, Mansoura University, Mansoura 35516, Egypt; hanaamaher1@yahoo.com (H.M.A.); sorriaeg@mans.edu.eg (M.S.S.); 6 Department of Basic Medical Sciences, College of Medicine, AlMaarefa University, Riyadh 13713, Saudi Arabia; msharbini@mcst.edu.sa; 7 Department of Anatomy, Faculty of Medicine, Mansoura University, Mansoura 35516, Egypt

**Keywords:** rapamycin, adipose-derived stem cells, cisplatin-nephrotoxicity, autophagy

## Abstract

Objective: Because the poor survival of transplanted cells in a hostile microenvironment limits stem cell therapy, in the current study, we investigated the effect of rapamycin (Rapa)-preactivated autophagy on the survival and homing of transplanted adipose mesenchymal stem cells (ADMSCs) in a rat model of cisplatin (Cis)-induced nephrotoxicity, as well as the possible role of the mTOR/AKT signaling pathway. Materials and methods: In vitro, ADMSCs isolated from rats were treated with 50 nmol/L rapamycin for 2 h, after which the cytoprotective and autophagy-inducing effects of Rapa were investigated. The cis-induced acute nephrotoxicity rat model was constructed in vivo. ADMSCs and Rapa-ADMSCs were administered into the tail vein before Cis therapy. At 3, 7, and 10 days after Cis injection, all animals were euthanized. The renal functions and morphology as well as autophagy response were assessed. Results: The pretreatment of cultured ADMSCs with Rapa caused a significant increase in autophagic activities and lysosome production of the cells, with a significant increase in the secretion of SDF-1, IL-10 and autophagy promoter LC3 and Beclin from these cells, while mTOR/AKT pathways were inhibited. In addition, the transplantation of Rapa-pretreated ADMSCs restored the kidney functions and morphology dramatically. Renal expression of SDF-1 and HIF1 was upregulated, while expression of IL-6, *NF-kB* and TGF-β1 was downregulated. Conclusions: We concluded that the preactivation of autophagy with Rapa improves the survival and differentiation of the transplanted ADMSCs by inhibiting the mTOR/AKT signaling pathway, which in turn could significantly attenuate the Cis-induced acute renal injury.

## 1. Introduction

Cisplatin (Cis), a platinum inorganic complex, is a powerful chemotherapeutic agent used to treat a variety of cancers, including testicular tumors, ovarian germ cell tumors, bladder cancer and adrenocortical carcinoma [1]. However, cisplatin’s cumulative nephrotoxicity and neurotoxicity limit its high-dose therapy [2]. Acute kidney damage (AKI) caused by cisplatin is linked to enhanced tubular epithelial cell proliferation and migration, as well as fibrosis and apoptosis [3]. As a result, to offset the renal damage caused by cisplatin, preventive measures are required. Mesenchymal stem cells (MSCs), also known as stromal stem cells, are a type of cell that has a wide range of therapeutics functions. MSCs have a recognized use in cell-based therapy given their ability to modulate inflammation and improve tissue regeneration and their low immunogenicity [4]. The potential applications of MSCs have been explored in a number of diseases associated with organ dysfunction and disorders of the immune system, such as acute liver failure and Crohn’s disease [5]. It has also been demonstrated that adipose-derived ADMSCs have potential therapeutic efficiency in chronic wounds, lower extremities ulcers and soft tissue defects such as burns and scars, craniofacial microsomia [6] and Corona virus disease 19 (COVID-19) [7,8,9].

Despite the evidence that cell therapy with MSCs contributes to the improvement of AKI, some challenges need to be overcome in order for such therapy to be successfully established, such as defining the best route of administration, establishing the number of cells per administration and the number of injections, finding the best strategy for MSCs to migrate to acute and chronic kidney injury, understanding the interaction between MSCs and other tissue cells, and determining the impact survival and incorporation of cells into the injured tissue [10]. Therefore, the efficient homing of MSCs towards tissue-specific conditions is one of the most important factors in successful stem therapy. The expression of stromal cell-derived factor-1 (SDF-1) or CXCL12 is well recognized to be elevated in injured organs such as the liver, brain, and kidney [11,12,13]. However, because only a small percentage of MSCs express CXCR4 (SDF-1 receptors) during in vitro development, their ability to respond to homing signals in injured areas could be limited [14]. Recent research studies have focused on developing novel ways for preconditioning MSCs during ex vivo expansion using hypoxic environments, pharmacologic agents and cytokines [15]. Autophagy modification in MSCs prior to transplantation has been shown to improve the survival and viability of engrafted MSCs as well as their pro-angiogenic, immunomodulatory and differentiation capability [16]. The induction of autophagy, according to a recent study, could improve MSC homing to damaged liver tissue and have a therapeutic impact in liver I/R injury [17]. Furthermore, autophagy is necessary for optimal proximal tubule function as well as protection from acute tubular injury [18].

Autophagy is the highly conserved fundamental cell biological pathway in which cytoplasmic materials are delivered to and degraded in the lysosome. Basal levels of autophagy are important in maintaining cellular homeostasis by elimination of damaged organelles, protein aggregates and turnover of long-lived proteins [19]. Multiple signaling pathways regulate autophagy; the mammalian target of rapamycin (mTOR) and AMP-activated rote in kinase (AMPK) pathways are the two major pathways that regulate autophagy in mammals [20]. Other pathways, such as the AKT/PKB, p52 and inositol pathways, are also important in autophagy modulation [21]. The dissociation of the mTORC1 and Beclin-1/class III phosphatidylinositol-3-kinase (PI3K) complex forms is induced by stressful conditions [22]. Rapamycin, a powerful autophagy activator, is a mTOR-specific inhibitor that inhibits the activation of p70S6K and the phosphorylation of 4EBP-1 in the cytoplasm to form a complex, blocking mTOR and its downstream signals. Rapamycin has been shown in the literature to reduce renal ischemia reperfusion injury by controlling mTOR and promoting autophagy [23]. The goal of this study is to see if inducing autophagy in ADMSCs with rapamycin could be a potential therapeutic method for treating the nephrotoxicity caused by cisplatin by inhibiting the mTOR/AKT pathway.

## 2. Materials and Methods

### 2.1. In Vitro Study of Pretreatment of ADMSCs with RAPAMYCIN

#### 2.1.1. Isolation, Culture and Characterization of ADMSCs

**Figure 1:**
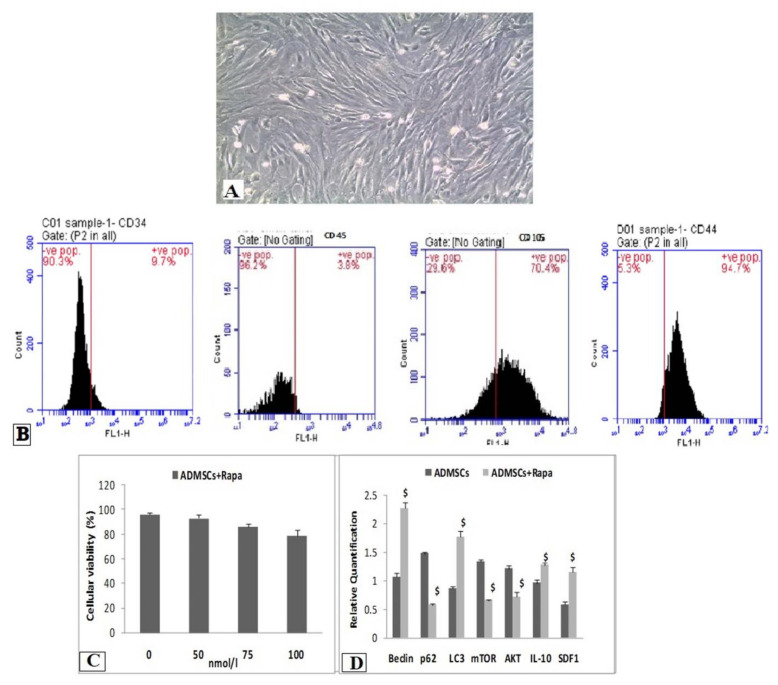
In vitro studies on Rapa-ADMSCs. (**A**) ADMSCs at passage 3 (400×), (**B**) flowcytometric analyses for the markers of ADMSCs (negative for CD34 and CD45 and positive for CD105 and CD44), (**C**) the effect of Rapa treatment on the viability of ADMSCs for 2 h in culture and (**D**) the expression rate of autophages, migration and pro-inflammatory genes for Rapa treated and untreated cells. ^$^ Significant vs. ADMSC group (*p* < 0.05).

ADMSCs were collected from the paragonadal fat of Sprague-Dawley rats testis, and details of its collection, culture and characterization are specified in our previous research work [24]. Figure 1A shows the cultured ADMSCs at passage 3. ADMSCs express on their surface the classical markers of mesenchymal stem cells, e.g., CD44, CD73, CD90, CD105 and CD166 [6]; thus, in the current study, we tested CD45, CD34, CD44 and CD105. The flow cytometric results demonstrated that the isolated ADMSCs were negative for CD34 (90.3%) and CD45 (96.2%) and positive for CD105 (70.4%) and CD44 (94.7%) (Figure 1B).

#### 2.1.2. In Vitro Pretreatment of ADMSCs with Rapamycin

ADMSCs were divided into two groups: (a) the untreated ADMSC group was used as a negative control; (b) the second group involved ADMSCs treated with 50 nmol/L of rapamycin in] the culture medium, and the cells were subsequently incubated for 2 h.

#### 2.1.3. Cytotoxicity Assay

Viability of the cells was assessed with MTT [3-(4,5-Dimethylthiazol-2-Yl)-2,5-Diphenyl-2HtetrazoliumBromide] assay. The cells were seeded in a 96-well plate, with three parallel wells for each group. For optimum Rapa concentration, the cells were treated with different doses of Rapa (50, 75 and 100 nmol/L) for 2 h. Then, 10 μL MTT (5 mg/mL; Dojindo, Kumamoto, Japan) was added to the wells, and the cells continued to be incubated for 2 h. After sucking up the supernatant, 100 μL DMSO was added to fully dissolve the generated formazan crystal. Absorbance values at 570 nm were measured using an Infinite 200 PRO Microplate Reader (Tecan, Mannedort, Switzerland).

#### 2.1.4. Gene Expression of Autophagy and Pro-Inflammatory Genes

The impact of Rapa treatment on gene expression of the pro-inflammatory marker (IL-10), autophagy markers; (LC3, mTOR, Beclin, P62 and AKT) and the migration marker (SDF1α) was analyzed using a Real-Time PCR system (Applied Biosystems, Waltham, MA, USA). Total RNA was extracted from MSC and Rapa-MSC groups using the RNeasy Mini Kit (Qiagen, 74104) according to the manufacture’s protocol. RNA was converted to cDNA by a QuantiTect Reverse Transcription Kit (Qiagen, 205310) according to the manufacture’s protocol. Quantitative RT-PCR analysis for mRNA was carried out with SYBER Green PCR Master Mix (Applied Biosystems, USA). The primers used and GABDH as PCR control were purchased from applied biosystem, USA. All the primers of the studied genes are listed in Table 1. The cycling parameters were as follows: initial denaturation at 95 °C for 10 min, followed by 40 cycles of denaturation 95 °C for 15 s, annealing at 60 °C for 1 min and extension at 72 °C for 1 min. Data analysis was carried out using Step one plus real-time PCR by the 2^−ΔΔCt^ method. GAPDH was used as a Housekeeping internal control gene.

#### 2.1.5. Immunohistochemical Staining of LC3 for Autophagic Structures

LC3 is expressed mainly on autophagosomes and is used as a specific marker for autophagic structures. After treatment with rapamycin for 2 h, the cells were fixed in 4% paraformaldehyde and permeabilized with 0.5% Triton X-100. Then, the cells were incubated with rabbit anti-LC3 antibody (1:100; Abcam, Cambridge, MA, USA) overnight at 4 °C. Biotinylated secondary antibody was added for 1 h at room temperature; the slides were washed with PBS, and DAB staining was applied to develop the reaction color. The tissue sections were counterstained with hematoxylin, dehydrated, mounted with a coverslip and observed by an Olympus BX51light microscopy.

#### 2.1.6. Transmission Electron Microscopy

Rapamycin-treated cells were fixed overnight in 3% glutaraldehyde and post-fixed in 1% osmic acid for 2 h. Subsequently, a series of dehydrations were performed. The cells were embedded in epoxy resin. Ultrathin sections were made by an ultramicrotome. After being stained with lead citrate and uranyl acetate, the autophagic structures in the cells were viewed using a transmission electron microscope (JEOL JEM-2100 at 160 KV, Electron Microscope Unit, Mansoura University, Mansoura, Egypt). The autophagic structures were examined in 200 cells for each group.

### 2.2. In Vivo Study of the Effect of ADMSCs Pretreated with Rapa on Renal I/R Injury

#### 2.2.1. Experimental Animals

The current study was carried out using 120 male Sprague-Dawley rats weighing ~200–250 g according to the Guide for the Care and Use of Laboratory Animals approved by the ethical committee, Urology and Nephrology Center, Mansoura, Egypt (ms/15.08.94.r4). All protocols were approved by the ethical committee of Mansoura University.

#### 2.2.2. Animal Groups

The rats were randomly allocated into 4 equal groups (each 30 rats) as follows: (i) control group: animals were injected intraperitoneally (i.p.) with 0.9% saline; (ii) cisplatin (Cis) group: rats were intraperitoneally injected with only one dose of cisplatin 6 mg/kg; (iii) ADMSC group: cisplatin group with administration of 1 × 10^6^ ADMSCs suspended in 0.08 mL complete medium i.v. in the tail vain; (iv) Rapa + ADMSC group: cisplatin group with the administration of 1 × 10^6^ ADMSCs pretreated with Rapa for 2 h. Rats were euthanized at 3, 7 and 10 days after Cis administration.

#### 2.2.3. Collection of Urine Blood and Tissue Samples

At 24 h before the sacrifice time, 24 h urine samples were collected from rats. Blood samples (1 mL) were collected from each group from the ophthalmic venous plexus using a fine-walled Pasteur pipette under light inhalation halothane anesthesia. Blood samples were centrifuged at 400× *g* for 10 min to obtain the sera that were stored at −20 °C until the time of analysis. For kidney samples, rats were euthanized using a large dose of Na thiopental (120 mg/kg), and the abdomen was rapidly opened. Right kidneys were harvested for the oxidative stress evaluation, while left kidneys were harvested and divided into two halves, one half was stored in liquid nitrogen for molecular measurements, and the second half was fixed in formalin 10% for histological and immunohistochemical examination.

#### 2.2.4. Measurement of Serum and Urine Creatinine and Serum Blood Urea Nitrogen (BUN) and Calculation of Creatinine Clearance

Measurements of serum and urine creatinine and blood urea nitrogen (BUN) with commercially available kits were performed according to the manufacturer’s instructions. The creatinine clearance was measured from the following equation.

Creatinine clearance = Urine creatinine (mg/dL) × urine volume (mL/24 h)/Serum creatinine (mg/dL) × 1440 (min) mL/min (1)

#### 2.2.5. Evaluation of the Renal Tissues Oxidative Stress State SOD, CAT, MDA and NO

To assess the oxidative stress state in kidney tissues, the activity of superoxide dismutase (SOD), catalase (CAT), malondialdehyde (MDA) and nitric oxide (NO) were measured in all studied groups at different times using commercial kits according to the manufacturer’s protocols (Biodiagnostic Co., Giza, Egypt).

#### 2.2.6. Gene Expression

The expression of Rapa-MSCs on pro-inflammatory (TGF-β1, IL-6 and NK-kB), angiogenesis (HIF-1α) and migration (SDF-1α) genes were assessed in kidney tissues using real-time PCR. Briefly, RNA was isolated from tissue samples of all groups, followed by cDNA synthesis, and the RT-PCR reaction was performed as described in our previous research work [24].

#### 2.2.7. Histopathological Examination

The histology of kidney was examined by H&E staining. Briefly, tissues were fixed with formalin, dehydrated, and embedded with paraffin. After being cut in 5-μm sections using a microtome (Leica Microsystems, Inc, Wetzlar, German), the kidney tissues were stained with H&E. The extent of damage was counted and scored in 10 randomly chosen fields (200×) as follows: 0, none; 1, <10%; 2, 10~25%; 3, 26~45%; 4, 46~75%; and 5, >75% [25].

#### 2.2.8. Western Blot Analysis

A total of 20 mg of kidney tissue was homogenized in 200 µL of RIPA buffer containing protease (Cell signaling technology, Danvers, MA, USA, 5871S) and phosphatase inhibitor cocktail for 20 min using an Ultrasonic Probe Sonicator on ice. The lysate was centrifuged at 4 °C for 20 min at 15,000× *g*. The protein concentration was determined by Pierce™ BCA Protein Assay Kit. Then, 30 µg/well was loaded to SDS-PAGE. Then, the protein was transferred to a 0.45-µm nitrocellulose membrane for 90 min at 90 V. The protein transfer was confirmed using Ponceau S stain. The membrane was blocked for 2 h with 3% BSA at room temperature, then incubated with primary antibodies against mTOR, AKT, pAKT or actin (Cell signaling technology) overnight at 4 °C. After washing the membrane, it was incubated with anti-rabbit HRP-conjugated secondary antibody for one hour at room temperature. After washing the membrane, the signal was detected using WesternBright™ ECL HRP substrate (Advansta, K-12045) and visualized by The ChemiDoc MP Imaging System (Bio-Rad, Hercules, CA, USA). The fold of change in protein level was calculated using GraphPad Prism 8 Software after normalization to the level of housekeeping protein β-actin.

#### 2.2.9. Immunohistochemical Examination of TGF-β1, SDF-1 and LC3

The primary antibodies of TGF-β1 (AB-246-NA), SDF-1α (MAB350) and LC3 (Cat#YPA1340) were purchased from Chongqing Biospes Co, Ltd., Chongqing, China, and were used for detection of these proteins in the renal tissue using immunohistochemical staining. Briefly, paraffin-embedded sections were subjected to antigen retrieval with 10 mM citrate buffer at pH 6 for 30 min. Subsequently, the expression of TGF-β1, SDF-1α and LC3 was detected at a dilution of 1:100 for TGF-β1, SDF-1α and 1:200 for LC3 for 1 h at room temperature, after which biotinylated secondary antibody was added for 1 h at room temperature. The sections were washed with PBS, and DAB staining was applied to develop the reaction color. The tissue sections were counterstained with hematoxylin, dehydrated, mounted with a coverslip and observed by an Olympus BX51light microscope.

#### 2.2.10. Statistical Analysis

Experimental data were presented as means ± SD. The two-tailed Student’s *t*-test and ANOVA with Tukey’s post hoc test were used to analyze the comparison between different groups, and *p* < 0.05 was set to indicate a significant difference.

## 3. Results

### 3.1. Effect of Rapa Pretreatment on ADMSC Viability

ADMSCs treated with different concentrations of Rapa for 2 h showed the highest cellular viability at concentration 50 nmol/L (Figure 1C).

### 3.2. Effect of Rapa Pretreatment on Autophagic, Inflammatory and Migratory Markers In Vitro

**Figure 2:**
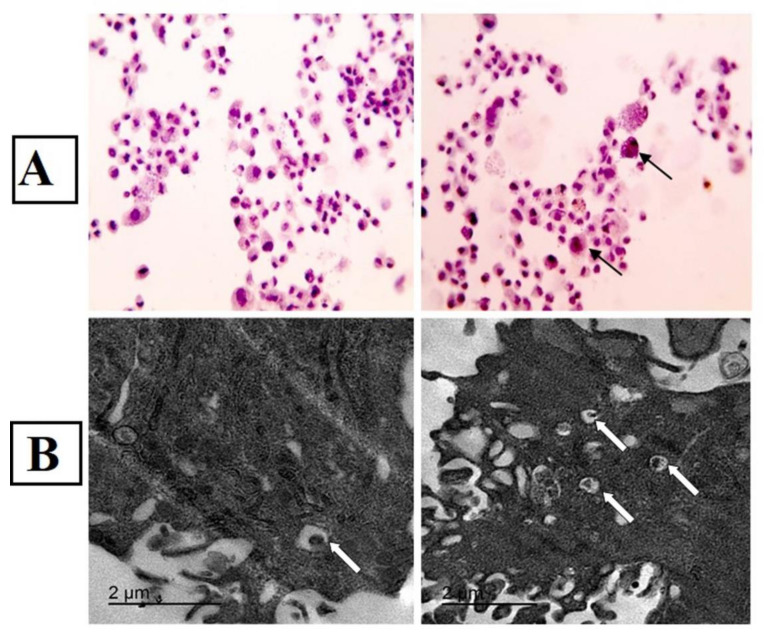
The effect of Rapa treatment on (**A**) LC3 expression using immunohistochemical studies: control ADMSCs against LC-3 show negative staining, and Rapa-treated ADMSCs against LC-3 show increased positive brown intracellular expression (arrows) ×400; (**B**) autophagic ultrastructures. Arrows indicate autophagosome.

The results of autophagy markers (Beclin, LC3, mTOR, p62 and AKT), the migration marker (SDF-1α) and the anti-inflammatory cytokine (IL-10) showed a significant increase in Rapa-ADMSCs compared to the untreated cells. In contrast, the expression of P62, mTOR and AKT markers were lower in Rapa-ADMSCs compared to the untreated cells (Figure 1D). Moreover, the result of immunohistochemistry showed that LC3-II expression in the cells treated with rapamycin for 2 h was significantly increased compared with the control group; the number of LC3-positive nuclear markers in the rapamycin group was greater (Figure 2A). Furthermore, transmission electron microscopy showed an increase in the autophagic ultrastructures in Rapa-ADMSCs compared to the untreated cells (Figure 2B).

### 3.3. Effect of Rapa-Treated ADMSCs on Kidney Functions

Rats treated with Cis showed higher levels of serum creatinine and BUN than the control group at the three different times (*p* < 0.01). ADMSC administration resulted in a significant decrease in serum creatinine and BUN compared to the Cis group (*p* < 0.01). Moreover, the ADMSCs pretreated with Rapa group revealed the lowest level of serum creatinine and BUN compared to the ADMSC group at 3, 7 and 10 days (*p* < 0.01). On the other hand, creatinine clearance was significantly reduced in the Cis group compared to the control group (*p* < 0.01) and significantly increased in the ADMSC and Rapa + ADMSC groups compared to the Cis group (*p* < 0.01). Furthermore, the Rapa + ADMSC group showed a greater reduction in creatinine clearance compared to ADMSC group at 7 and 10 days (*p* < 0.01) (Table 2).

### 3.4. Effect of Rapa-Treated ADMSCs on Oxidative Stress Markers (NO, MDA, CAT and SOD) in Kidney Tissues

The Cis group showed a significant decrease in CAT and SOD activities compared to the control group, whereas their activities were significantly increased with the administration of ADMSCs and showed a greater increase in the Rapa + ADMSC group at 3, 7 and 10 days (*p* < 0.01). On the other hand, MDA and NO levels were significantly higher in the Cis group compared to the control (*p* < 0.01), while the ADMSC and Rapa + ADMSC groups showed a significant decrease in MDA and NO levels compared to the Cis group (*p* < 0.01). Moreover, Rapa + ADMSCs revealed a greater reduction in MDA levels at 3, 7 and 10 days and in NO levels at 7 and 10 days than the ADMSC group (*p* < 0.01) (Table 3).

### 3.5. Effect of Rapa-Treated ADMSCs on the Expression of Pro-Inflammatory, Angiogenesis and Migration Markers

**Figure 3:**
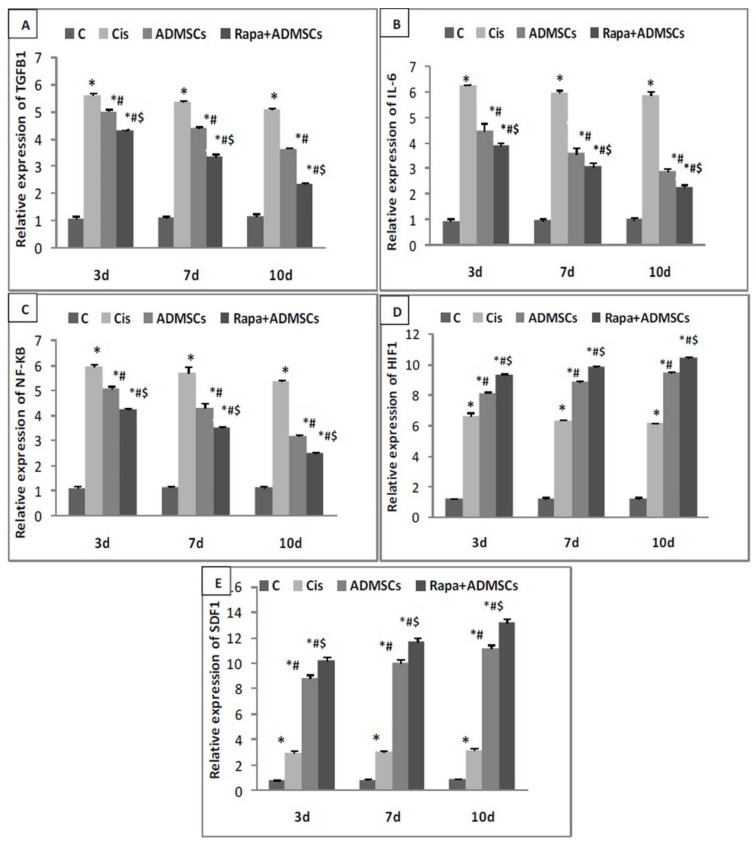
The impact of Rapa-ADMSC administration on (**A**) TGF-β1, (**B**) IL-6, (**C**) NF-kB, (**D**) HIF-1α and (**E**) SDF-1α expression at 3, 7 and 10 days after cisplatin injection. * Significant vs. control, # significant vs. Cis group and $ significant vs. ADMSC group.

The ameliorating effect of Rapa-ADMSCs on the gene expression of TGF-β1, IL-6, NF-kB, HIF-1α and SDF-1α was studied in the renal tissue samples of all experimental groups using real-time PCR. The expression of pro-inflammatory TGF-β1 and IL-6 in kidney tissues was significantly higher in the Cis group compared to the control group at 3, 7 and 10 days (*p* < 0.01), while their expressions were significantly lower in ADMSCs and Rapa + ADMSCs compared to the Cis group at different times (*p* < 0.01). Moreover, their expressions were significantly lower in the Rapa + ADMSC group compared to the ADMSC group (*p* < 0.01) (Figure 3A,B).

Additionally, the expression of apoptotic NF_K_B in kidney tissues was significantly higher in the Cis group compared to control group at 3, 7 and 10 days (*p* < 0.01), while its expression was significantly lower in ADMSCs and Rapa + ADMSCs compared to the Cis group at different times (*p* < 0.01). Furthermore, their expressions were significantly lower in the Rapa + ADMSC group compared to ADMSCs group (*p* < 0.01) (Figure 3C).

On the other hand, the expression of angiogenesis marker HIF-1α and migration marker SDF-1α was significantly higher in the Cis group compared to the control group at 3, 7 and 10 days (*p* < 0.01). Moreover, their expression showed a greater increase in ADMSCs compared to the Cis group at the three-time intervals (*p* < 0.01). Furthermore, Rapa + ADMSCs showed the greatest increase in HIF-1α and SDF-1α gene expression compared to the ADMSC group (*p* < 0.01) at 3, 7 and 10 days (Figure 3D,F).

### 3.6. Effect of Rapa-Treated ADMSCs on the Expression of mTOR and AKT

**Figure 4:**
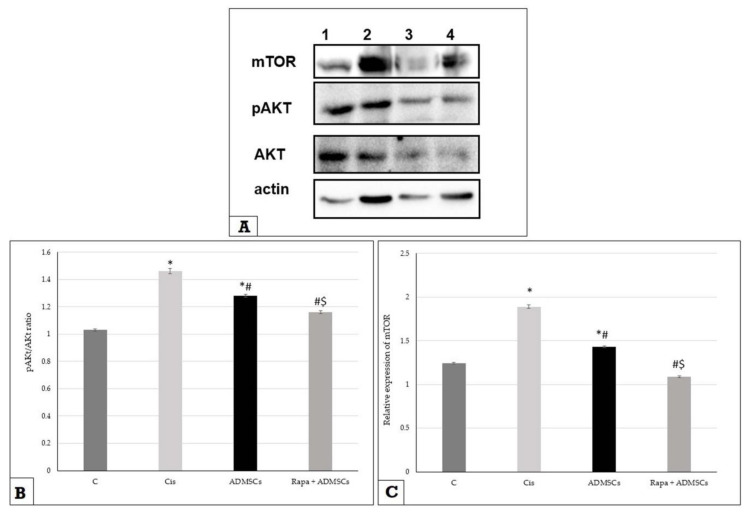
The impact of Rapa-ADMSC administration on Akt/pAkt and mTOR expressions at 10 days after cisplatin injection. (**A**) the products of western blotting of the tested genes, lane 1 = control group, lane 2 = Cis group, lane 3 = Rapa + ADMSC group and lane 4 = ADMSC group, (**B**) pAkt/Akt ratio and (**C**) mTOR expression in different studied groups. * Significant vs. control, # significant vs. Cis group and $ significant vs. ADMSC group.

Figure 4A is a representative sample for the products of western blotting for pAkt, Akt, mTOR and β-actin at day 10 after Cis administration. The expression of mTOR and phosphorylated AKT at the level of proteins at day 10 was significantly higher in the Cis group compared to the control group (*p* < 0.01), while their expressions were significantly lower in ADMSCs and Rapa + ADMSCs compared to the Cis group at different times (*p* < 0.01) (Figure 4B,C).

### 3.7. Effect of Rapa-Treated ADMSCs on Renal Histopathological Changes

**Figure 5:**
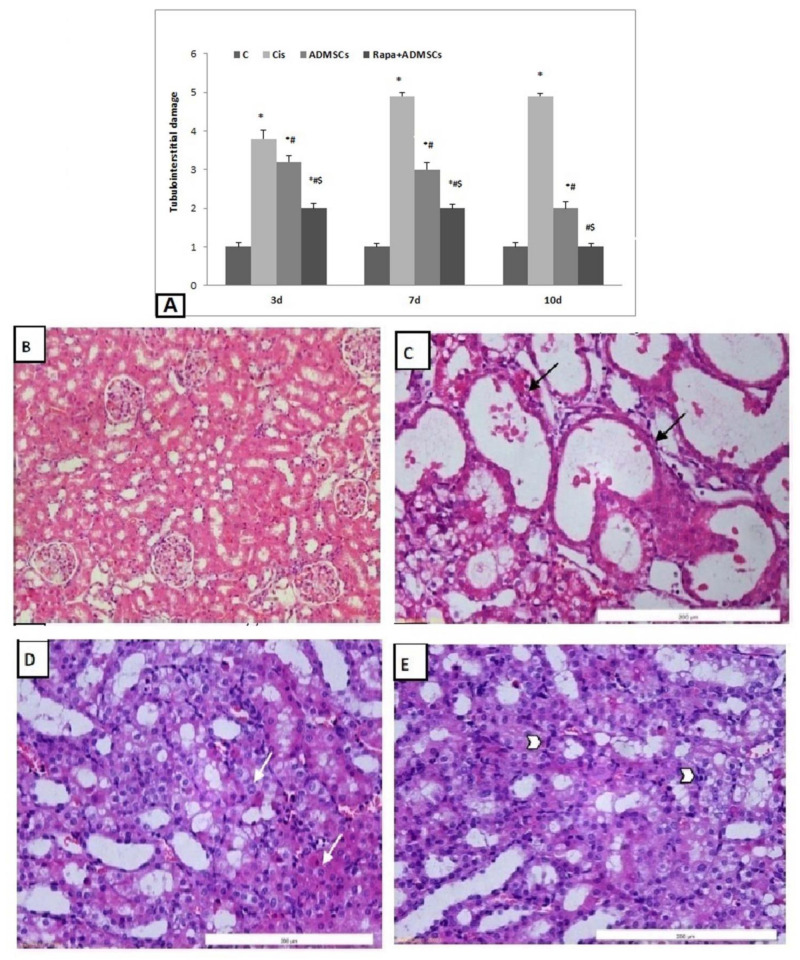
H&E staining showing the structure of renal tissue and damage score at different time intervals, showing (**A**) effects of MSCs pretreated with rapamycin on tubulointerstitial damage score at different time intervals, (**B**) kidney specimens with normal kidney structure (control group at 7 days), (**C**) apoptotic cells with dilated irregular tubules (black arrow) (Cisplatin 7 days), (**D**) prominent nucleoli with few apoptotic cells (white arrow) (ADMSC group at 7 days), (**E**) regeneration in the form of mitotic figures (arrow head) (Rapa + ADMSC group at 7 days). * Significant vs. control, # significant vs. Cis group and $ significant vs. ADMSC group.

The Cis group showed a significant increase in the histopathological damage score compared to the control group (*p* < 0.01); this score was significantly attenuated in the ADMSC and Rapa + ADMSC groups at the three different times (Figure 5A). Normal kidney morphology was shown in the control group (Figure 5B), whereas the Cis group showed severe damage in the form of irregular dilated tubules, apoptotic cells and interstitial hemorrhage (Figure 5C). Treatment with ADMSCs and Rapa + ADMSCs showed low apoptotic cells and degree of regeneration in the form of mitotic figures and prominent nuclei (Figure 5D,E, respectively).

### 3.8. Effect of Rapa-Treated ADMSCs on the Protein Expression of TGF-β1, SDF-1α and LC3

**Figure 6:**
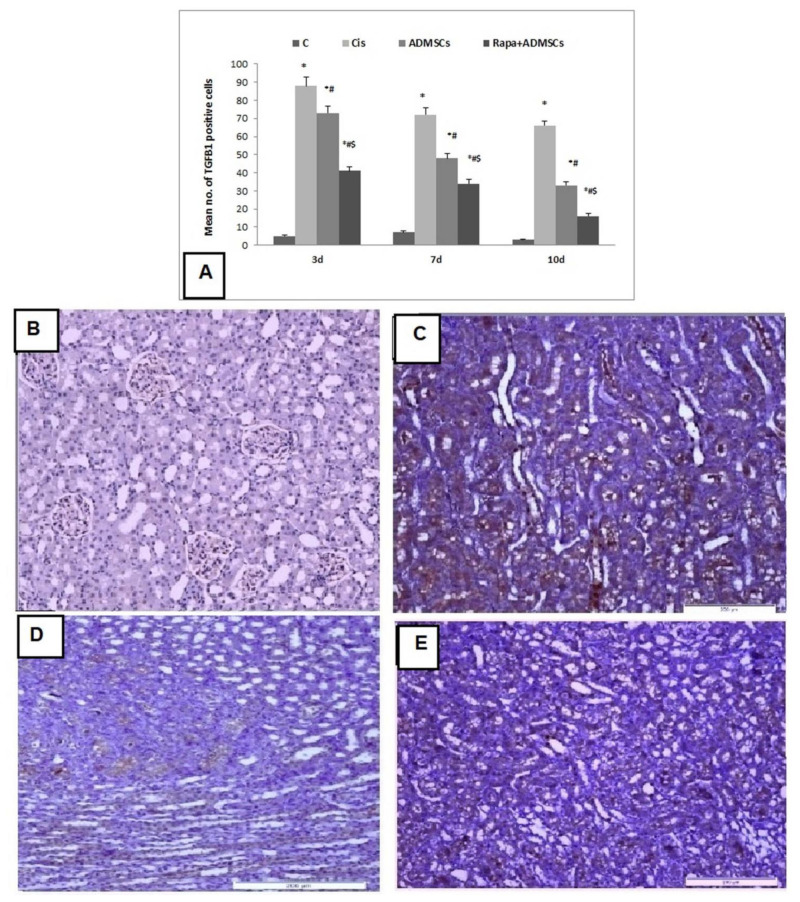
Immunohistochemical analysis of TGF-β1 expression in kidney tissues 400×. (**A**) Immunoscore of TGF-β1, (**B**) negative expression in control group, (**C**) marked expression in cisplatin group, (**D**) moderate expression in ADMSC group, (**E**) mild expression in Rapa + ADMSC group. * Significant vs. control, # significant vs. Cis group and $ significant vs. ADMSC group.

The score of TGF-β1 expression was significantly higher in the Cis group than the control group (*p* < 0.01), and its expression became lower with ADMSC and Rapa + ADMSC groups (Figure 6A). The control group showed negative expression of TGF-β1 (Figure 6B), while the Cis group showed marked expression of TGF-β1 (Figure 6C). ADMSC treatment showed moderate expression of TGFβ1 (Figure 6D), and mild expression was observed in the Rapa + ADMSC group (Figure 6E).

**Figure 7:**
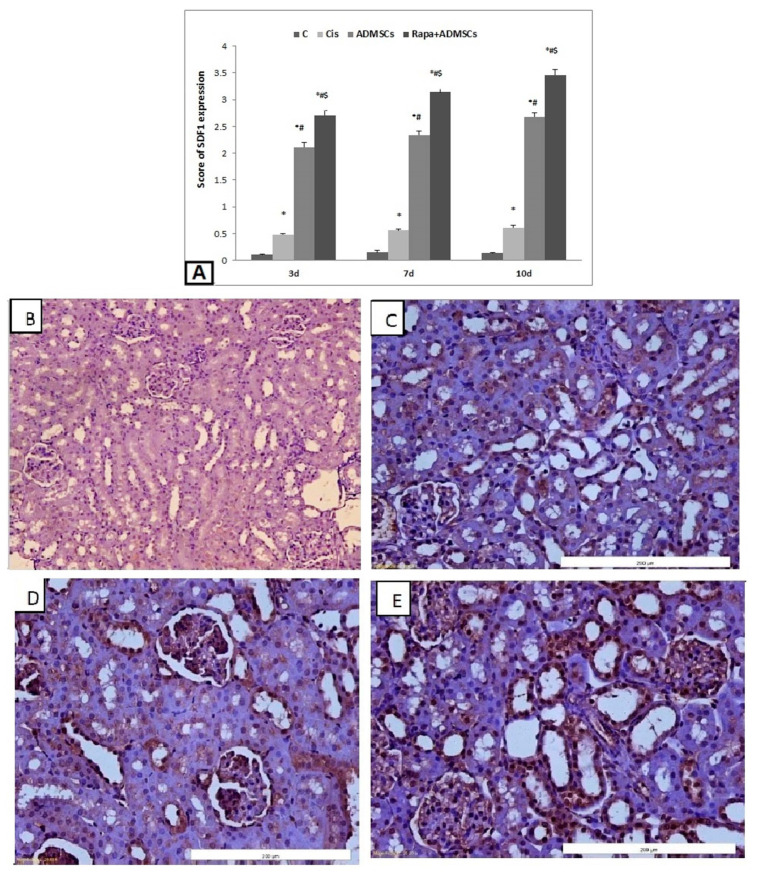
Immunohistochemical analysis of SDF1α expression in kidney tissues 400×. (**A**) Immunoscore of SDF1α, (**B**) negative expression in control group, (**C**) mild expression in cisplatin group, (**D**) moderate expression in ADMSC group, (**E**) marked expression in Rapa + ADMSC group. * Significant vs. control, # significant vs. Cis group and $ significant vs. ADMSCs group.

On the other hand, the score of SDF-1α expression was significantly higher in the Cis group than the control group (*p* < 0.01), and its expression became higher in the ADMSC and Rapa + ADMSC groups (Figure 7A). The control group showed negative expression of SDF-1α (Figure 7B), which become mild with the Cis group (Figure 7C). Compared to the Cis group, SDF-1α expression was higher in ADMSCs (Figure 7D), and a marked increase in expression was noticed in the Rapa + ADMSCs group (Figure 7E).

**Figure 8:**
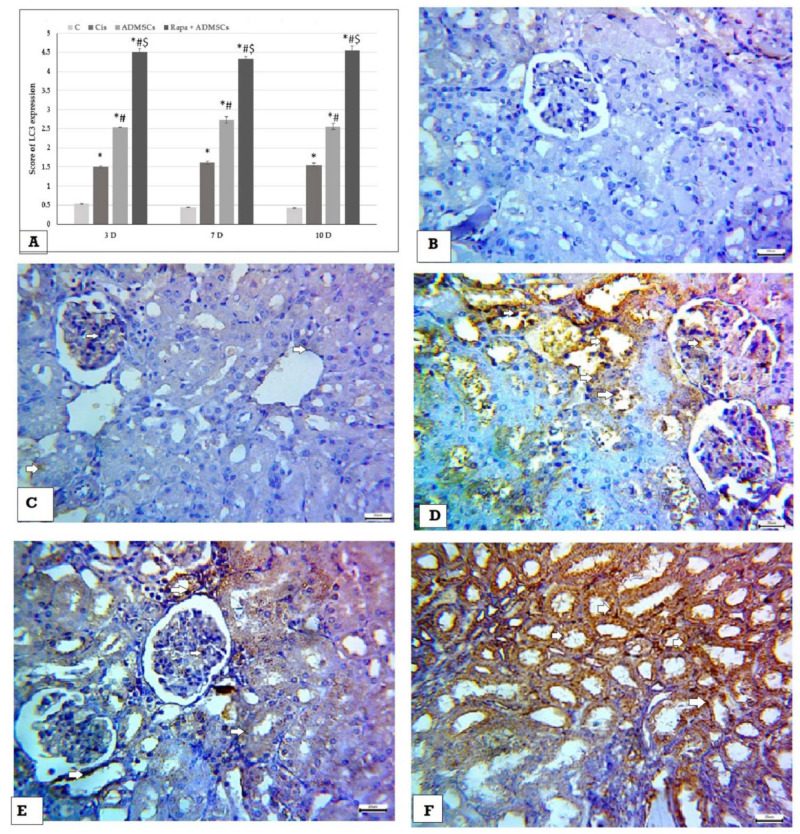
Immunohistochemical analysis of LC3 expression in kidney tissues 400× (**A**) Immunoscore of LC3, (**B**) negative expression in control group, (**C**) mild cytoplasmic expression (white arrows) in cisplatin group, (**D**) moderate cytoplasmic expression (white arrows) in ADMSC group, (**E**,**F**) marked cytoplasmic expression (white arrows) in Rapa + ADMSC group. * Significant vs. control, # significant vs. Cis group and $ significant vs. ADMSCs group.

In addition, the score of LC3 expression was significantly higher in the Cis group than the control group (*p* < 0.01), and its expression became higher in the ADMSC and Rapa + ADMSC groups (Figure 8A). The control group showed negative expression of LC3 (Figure 8B), which become mild in the Cis group (Figure 8C). Compared to the Cis group, LC3 expression was higher in ADMSCs (Figure 8D), and a marked increase in expression was noticed in the Rapa + ADMSC group (Figure 8E,F).

## 4. Discussion

The main findings of the present study can be summarized as follows: (i) pretreatment of cultured ADMSCs with Rapa resulted in a significant increase in cell viability, autophagic structures by EM, expression of autophagic markers such as Beclin and LC3, anti-inflammatory cytokine IL-10 expression, and chemoattractant gene SDF-1 expression, with a significant reduction in the expression of other autophagic markers such as mTOR, p62 and AKT; (ii) treatment with ADMSCs pretreated with Rapa offered a more powerful renoprotective effect against Cis-induced nephrotoxicity than ADMSCs alone; and (iii) the renoprotective impact of ADMSCs was related with overexpression of HF-1α and SDF-1 and downregulation of profibrotic cytokines (TGF-β1), inflammatory cytokines (IL6 and NF-kB) and oxidative stress.

The first aim of this work was to see how Rapa pretreatment affected the expression of inflammatory and autophagic mediators in cultured ADMSCs. Autophagy has been shown to be important for the physiological function of podocytes in the kidney [26] as well as the protection of renal tubular cells against ischemia and nephrotoxicants such as cisplatin renal damage [1,27]. In addition, recent research has found that autophagy plays an important role in MSC-promoted tissue regeneration [28]. The mTOR gene is involved in protein synthesis and autophagy regulation [29]. The mTORC1 activation begins with the phosphorylation of PI3K to generate PIP3, which then activates the downstream protein Akt, triggering a cascade reaction that eventually activates mTORC1 [30]. Autophagy is negatively regulated by mTOR, which is inhibited by Rapa and helps in autophagy induction [31]. In line with these findings, the current investigation found that, via inhibiting mTOR phosphorylation, AKT and p62, ADMSC pretreatment with Rapa boosted the expression of the autophagic marker protein LC3II and beclin. Moreover, LC3-positive puncta and autophagic ultrastructures were increased. LC3 includes two forms: LC3-I is cytosolic, while LC3-II is expressed specifically on the membrane of the autophagic structures. Therefore, LC3-II expression provides an accurate measure of autophagic flux [32]. Interestingly, the current study was the first to detect the effect of ADMSC pretreatment with Rapa for 2 h on inducting autophagy in cells, increasing the cellular viability, the expression of the anti-inflammatory marker IL-10 and the migration capacity of the cells. We found that Rapa pretreatment increased the percentage of viable cells and anti-inflammatory properties and it improved the migration capacity of the cells by increasing the expression of SDF-1α; thus, it could enhance the homing of ADMSCs towards the injured tissue.

The current study’s second aim was to see if Rapa pretreatment on ADMSCs could protect rats against Cis-induced kidney injury. Cis administration in rats resulted in a significant increase in serum creatinine and BUN, as well as a significant reduction in creatinine clearance and tubulointerstitial damage in kidney tissues in the form of irregular dilated tubules, apoptosis, and infiltration of leukocytes, indicating deterioration of renal functions and morphology. Previous investigations have found that Cis has a negative effect on kidney function and shape, which is consistent with the current findings [33,34]. In addition, the present study found that pretreatment with either ADMSCs alone or ADMSCs pretreated with Rapa significantly attenuated the Cis-induced impairment in kidney functions and morphology, suggesting and confirming the efficiency of ADMSCs in treating nephrotoxicity, as reported in previous studies [24,35,36,37,38,39,40,41,42]. Furthermore, the ADMSCs pretreated with Rapa offered more renoprotective effect again Cis-induced renal injury than ADMSCs alone, suggesting the enhancing effect of Rapa on the therapeutic effects of ADMSCs. These findings support Rapa’s renoprotective impact in animal models of chemically-induced kidney injury, such as iodixanol-induced renal injury in diabetic rats [43], as well as Rapa’s ability to augment stem cell therapy [44].

The production of reactive oxygen species (ROS), accumulation of lipid peroxidation products (oxidative stress) in kidneys, and suppression of antioxidant systems are thought to be major mechanisms of Cis-induced renal injury; thus, in the current study, we investigated the role of oxidative stress in Cis-induced renal damage and the renoprotective effects of ADMSCs pretreated with Rapa. Cis impairs the respiratory chain and antioxidant defence systems, including SOD and CAT, causing mitochondrial dysfunction [45]. This could explain the significant reduction of the antioxidants, including SOD, GSH and CAT, with the significant increase in lipid peroxidation products (MDA) and nitric oxide (NO) levels in kidney tissues observed in the current study. Additionally, the current study found that treatment with either ADMSCs alone or ADMSCs pretreated with Rapa reduced MDA and NO levels while increasing SOD and CAT activity, confirming ADMSCs’ ability to protect cell membrane integrity from oxidative stress damage and increase antioxidant enzyme activities [46]. Furthermore, ADMSCs that had been pretreated with Rapa had a stronger antioxidant impact than ADMSCs that had not been pretreated. Wang et al. [33,34] found that Rapa inhibits ROS and promotes autophagy in renal tissues, demonstrating that its mechanism of action is connected to the inhibition of the mTOR/p70S6K signaling pathway.

The anti-inflammatory and antifibrotic impact of Rapa on ADMSCs in renal injury induced by Cis was evaluated. Transforming growth factor (TGF-β1) was reported to stimulate extracellular matrix protein production and deposition, leading to glomerulosclerosis and tubulointerstitial fibrosis [47]. In our study, the TGF-β1 gene and protein levels in renal tissues of the Cis group increased after 3, 7 and 10 days after injection, and its expression was downregulated after administration of ADMSCs. Furthermore, ADMSCs pretreated with Rapa showed greater reduction in *TGF-β1* gene and protein levels than ADMSCs. Likewise, the pro-inflammatory cytokine IL-6 was significantly reduced by ADMSCs treatment and become more significant with pretreatment with rapamycin, confirming the role of rapamycin in increasing the anti-inflammatory and antifibrotic efficacy of stem cells. Additionally, the role of angiogenesis as a potential mechanism for the cytoprotective effect of stem cell therapy is discussed in several studies [24,48]. Hypoxia-inducible factor 1-α (HIF-1α) is a transcription factor that regulates the expression of several genes involved in angiogenesis cell proliferation and survival [24]. Our study showed high mRNA expression of HIF-1α in the cisplatin group, which was higher in ADMSCs compared to the cisplatin group and reached the maximum increase with the rapamycin pretreatment, suggests this combination enhanced the process of angiogenesis. In agreement with these findings, Li et al. [44] found that paracrine of rapamycin-pretreated MSCs and the myocardium may account for myocardial regeneration and angiogenesis after transplantation.

Zhou et al. [7] examined the impact of intravenous administration of mesenchymal stem cells (MSCs) in seven patients with Coronavirus Disease 2019 (COVID-19) and reported an interesting significant improvement in their pulmonary functional activities after stem cell administration. This improvement could be attributed to the immune-modulatory and anti-inflammatory effects of MSCs, as these cells release many paracrine factors, which interact with immune cells, resulting in immunomodulation and inflammation decrease [8,9]. These effects were confirmed by the increased amount of peripheral lymphocytes, the decline in the C-reactive protein, and waning of over-activated cytokine-secreting immune cells (CXCR3 + CD4+ T cells, CXCR3 + CD8+ T cells, and CXCR3 + NK cells) into the blood of treated patients, by a mean 4.5 days after the intravenous infusion [7].

On the other hand, Song et al. [49] conducted a study in adriamycin-induced nephropathy rats and reported that MSCs can attenuate the nephropathy by diminishing oxidative stress and inhibiting the inflammation via downregulation of NF*κ*B. These findings are consistent with our findings, which revealed a considerable increase in NF*κ*B expression in the cisplatin group, which was reduced after treatment with ADMSCs. Furthermore, pretreatment with rapamycin resulted in a greater decrease in NF*κ*B mRNA expression, implying that it plays a role in improving cell therapy’s anti-apoptotic efficiency. Yang et al. [50] discovered that SDF-1α enhanced migration of dental pulp stem cells by activating autophagy, which was previously shown to impact macrophage migratory abilities. In this study, we first discovered that rapamycin increased SDF-1α expression on ADMSCs in vitro. In comparison to the ADMSC group, SDF-1α mRNA and protein expression were shown to be higher in renal tissue in ADMSCs pretreated with Rapa. The current study has some limitations, such as the fact that we did not assess the tested genes at the protein level but rather at the mRNA level. This is something that should be looked at further.

## 5. Conclusions

In the current study, we found that rapamycin can activate autophagy in ADMSCs, which increases cell migration and anti-inflammatory activity. Furthermore, rapamycin improved the therapeutic capacity of ADMSCs by enhancing cell homing to the injured kidney, promoting angiogenesis, and reducing pro-inflammatory cytokine production. Microvesicles secreted by ADMSCs may trigger autophagy, which aids in the recovery of injured kidneys; however, the mechanism of action of such microvesicles must be investigated.

## Figures and Tables

**Table 1:** Primers sequences for Real-Time PCR.

Genes	Sequence (5′–3′)	Accession Number
*LC3*	F:CCAGGAGGAAGAAGGCTTGG R:GAGTGGAAGATGTCCGGCTC	NM_022867.2
*P62*	F:TGCTCCATCAGAGGATCCCA R:TTTCTGCAGAGGTGGGTGTC	NM_175843.4
*Beclin*	F:CTCGTCAAGGCGTCACTTCT R:CCTCCATTCTTTAGGCCCCG	NM_053739.2
*IL-10*	F:GAAAAATTGAACCACCCGGCA R:TTCCAAGGAGTTGCTCCCGT	NM_012854.2
*AKT*	F:GAGGAGGAGACGATGGACTTC R:GGCATAGTAGCGACCTGTGG	NM_033230.3
*mTOR*	F:TTGTGTCCTGCTGGTCTGAAC R:GCTCTTTGTAGTGTAGTGCTTTGG	NM_019906.2
*SDF1α*	F:GAGCCATGTCGCCAGAGCCAAC R:CACACCTCTCACATCTTGAGCCTCT	NM_001033882.1
*TGF-β1*	F:CACTCCCGTGGCTTCTAGTG R:GGAC TGGCGAGCCTTAGTTT	NM_021578.2
*HIF-1α*	F:TGCTTGGTGCTGATTTGTGA R:GGTCAGATGATCAGAGTCCA	NM_024359.1
*IL-6*	F:GCCCTTCAGGAACAGCTATGA R:TGTCAACAACATCAGTCCCAAGA	NM_012589.2
*NF-κB*	F:GGACAGCACCACCTACGATG R:CTGGATCACTTCAATGGCCTC	NM_001276711.1
*GAPDH*	F:AGACAGCCGCATCTTCTTGT R:TTCCCATTCTCAGCCTTGAC	NM_017008.4

**Table 2:** Effects of MSCs pretreated with rapamycin on kidney functions (serum creatinine and BUN) at 3, 7 and 10 days after cisplatin induction.

Groups	3 Days (*n* = 10)	7 Days (*n* = 10)	10 Days (*n* = 10)
**Serum Creatinine (mg/dL)**			
Control	0.49 ± 0.02	0.48 ± 0.02	0.47 ± 0.02
Cis	2.07 ± 0.14 *	1.7 ± 0.08 *	1.18 ± 0.06 *
ADMSCs	1.7 ± 0.06 *^#^	1.38 ± 0.05 *^#^	1.02 ± 0.06 *^#^
ADMSCs + Rapa	1.55 ± 0.05 *^#$^	1.01 ± 0.06 *^#$^	0.78 ± 0.05 *^#$^
**BUN (mg/dL)**			
Control	20.79 ± 1.57	21.11 ± 1.08	21.33 ± 0.76
Cis	70.1 ± 1.63 *	63.68 ± 6.13 *	55.04 ± 3.32 *
ADMSCs	54.95 ± 3.75 *^#^	42.18 ± 3.67 *#	33.31 ± 3.52 *^#^
ADMSCs + Rapa	41.56 ± 4.2 *^#$^	31.28 ± 3.38 *#$	28.69 ± 2.97 *^#$^
**Creatinine Clearance (mL/min)**			
Control	1.38 ± 0.04	1.37 ± 0.04	1.41 ± 0.03
Cis	0.09 ± 0.02 *	0.13 ± 0.04 *	0.18 ± 0.05 *
ADMSCs	0.43 ± 0.08 *^#^	0.6 ± 0.05 *#	0.91 ± 0.11 *^#^
ADMSCs + Rapa	0.63 ± 0.09 *^#^	0.94 ± 0.42 *^#$^	1.31 ± 0.08 ^#$^

All data are expressed as mean ± SD. One-way ANOVA with Tukey post hoc test. * Significant vs. control group, ^#^ significant vs. Cisplatin group, ^$^ significant vs. ADMSC group.

**Table 3:** Effects of MSCs pretreated with rapamycin on oxidative stress/antioxidant levels at 3, 7 and 10 days after cisplatin induction.

Groups	3 Days (*n* = 10)	7 Days (*n* = 10)	10 Days (*n* = 10)
**Superoxide Dismutase (SOD) Activity (U/g kidney tissue)**			
Control	203.8 ± 4.57	205.4 ± 4.76	208.7 ± 5.68
Cis	95.57 ± 12.2 *	106 ± 7.68 *	114.9 ± 8.95 *
ADMSCs	116.6 ± 6.58 *^#^	138.4 ± 6.9 *^#^	156.9 ± 12.95 *^#^
MSCs + Rapa	133.2 ± 6.85 *^#$^	164.4 ± 7 *^#$^	177.9 ± 7.47 *^#$^
**Catalase (CAT) Enzyme Activity (U/g kidney tissue)**			
Control	5.02 ± 0.05	5.11 ± 0.08	4.99 ± 0.1
Cis	1.36 ± 0.1 *	1.59 ± 0.08 *	1.72 ± 0.07 *
ADMSCs	1.93 ± 0.07 *^#^	2.3 ± 0.05 *^#^	3.01 ± 0.11 *^#^
ADMSCs+ Rapa	3.16 ± 0.14 *^#$^	3.97 ± 0.05 *^#$^	4.1 ± 0.16 *^#$^
**Malondialdehyde (MDA) (nmol/g kidney tissue)**			
Control	19.44 ± 1.17	18.12 ± 1.32	20.61 ± 1.65
Cis	67.37 ± 5.91 *	71.21 ± 2.95 *	74.55 ± 3.56 *
ADMSCs	48.08 ± 4.69 *^#^	41.28 ± 2.8 *^#^	34.38 ± 3.97 *^#^
ADMSCs + Rapa	40.91 ± 2.07 ^*#$^	32.31 ± 4.46 ^*#$^	25.17 ± 2.91 ^#$^
**Nitric Oxide (NO) (nmol/g kidney tissue)**			
Control	9.71 ± 1.39	10.86 ± 1.04	11.04 ± 1.78
Cis	57.29 ± 4.14 ^*^	51.43 ± 2.46 *	48.9 ± 2.2 *
ADMSCs	43.16 ± 3.87 *^#^	35.59 ± 3.87 *^#^	28.95 ± 2.38 *^#^
ADMSCs + Rapa	38.87 ± 2.65 *^#^	27.3 ± 2.93 *^#$^	19.67 ± 2.06 *^#$^

All data are expressed as mean ± SD. One-way ANOVA with Tukey post hoc test. * Significant vs. control group, ^#^ significant vs. Cisplatin group, ^$^ significant vs. ADMSC group.

## Data Availability

All raw data will be available on request.
